# Magneto‐Electrodeposition and Field‐Enhanced Ion Transport in Zn‐MnO_2_ Batteries

**DOI:** 10.1002/smll.74574

**Published:** 2026-07-19

**Authors:** Pedaballi Sireesha, William T. McLeod, Kaylie A. McCracken, Jeffrey G. Bell

**Affiliations:** ^1^ Department of Chemistry Washington State University Pullman Washington USA; ^2^ The Gene and Linda Voiland School of Chemical Engineering and Bioengineering Washington State University Pullman Washington USA

**Keywords:** aqueous Zn‐MnO_2_ batteries, charge‐transfer resistance, ion‐transport enhancement, Lorentz‐forces, magneto electrodeposition

## Abstract

Aqueous zinc‐ion batteries (AZIBs) are promising alternatives to lithium‐ion systems for safe and low‐cost energy storage; however, their practical application is limited by poor cyclability, cathode degradation, and sluggish kinetics. Here, we introduce a magnetic‐field‐assisted electrodeposition strategy, termed magneto‐electrodeposition (MED), to fabricate MnO_2_ cathodes. The applied magnetic field induces Lorentz‐force‐driven magnetohydrodynamics, enhancing Mn^2+^ ion transport and enabling the formation of uniform and robust MnO_2_ coatings with enhanced δ‐MnO_2_ character. Structural analyses confirm that, through the MED approach, MnO_2_ depositions with higher Mn content (14.6%) and mass loading (26.6%) are obtained compared to conventional electrodeposited cathodes. Electrochemical characterization revealed improved charge‐transfer kinetics, with ∼95% reduced interfacial resistance (R_ct_). The optimized MED MnO_2_ cathode delivered an initial areal capacity of 0.62 mAh cm^−^^2^, nearly twice the conventionally electrodeposited cathode, with 73% capacity retention after 100 cycles. Introducing an internal magnetic field during operation further enhances performance, with 10 mT being optimal for improving Zn^2^^+^ transport. Consequently, the Zn‐MnO_2_ cell employing the MED cathode delivers stable cycling for ∼315 cycles, outperforming the conventionally electrodeposited cathode, which fails after ∼130 cycles. This work demonstrates that coupling magneto‐electrodeposition with in‐operando magnetic‐field‐assistance provides a scalable strategy to engineer cathodes and regulate ion transport for high‐performance AZIBs.

## Graphical abstract

Magneto‐electrodeposition (MED) strategy to improve the deposition uniformity and subsequent performance of MnO_2_ cathodes for aqueous Zn‐MnO_2_ batteries. Internal magnetic fields operating during battery cycling stabilize Zn anode and promote enhanced ion transportation, leading to enhanced initial areal capacities.

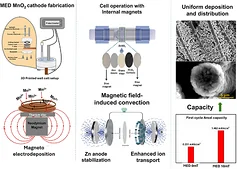

## 1 Introduction

The increasing global demand for safe, sustainable, and cost‐effective energy storage technologies has catalyzed research into aqueous battery systems, especially aqueous zinc‐ion batteries (AZIBs) [1, 2, 3]. AZIBs offer several advantages over lithium‐ion batteries, including intrinsic safety, environmental benignity, low manufacturing cost, and the high theoretical capacity (820 mAh g^−^^1^) of zinc anodes [3, 4, 5]. However, their practical deployment is limited by the lack of stable, high‐capacity cathode materials that can operate reliably in aqueous environments [6, 7]. Among various candidates, manganese dioxide (MnO_2_) is particularly promising owing to its earth abundance, low toxicity, polymorphism, and high theoretical capacity (∼308 mAh g^−^^1^) [8, 9, 10]. MnO_2_ is paramagnetic, so external magnetic fields can influence its structure, morphology, and ion transport properties. While high‐field effects (2–8 T) have been studied, the impact of low magnetic fields remains largely unexplored despite its importance in energy storage applications [11, 12]. The diverse MnO_2_ crystal structures α, β, γ, and δ phases enable various Zn^2^^+^ intercalation mechanisms, with δ‐MnO_2_ being particularly attractive because of its layered structure and large interlayer spacing that facilitate rapid and reversible Zn^2^^+^ insertion/extraction [13, 14, 15]. Despite these benefits, MnO_2_‐based cathodes often face challenges such as poor electronic conductivity, limited structural stability, and rapid dissolution in aqueous electrolytes, all of which lead to poor cycling performance [13, 16]. To address these limitations, various strategies, including doping [17, 18, 19], composite formation with carbonaceous materials [20], and morphological engineering (e.g., nanowires, nanoflowers, hollow structures, nanosheets, etc.) have been explored [21, 22, 23, 24]. Several studies have been reported using hydrothermal [25] and sol‐gel methods [26] to synthesize MnO_2_ for electrochemical applications. Developing a simple and ambient temperature synthetic route for MnO_2_ nanomaterials is critically important [27]. Among the various fabrication techniques, electrodeposition has emerged as a simple and scalable approach for synthesizing MnO_2_ electrodes with good contact between the active material and the current collector, although uniform distribution and dimensional control of nanoscale material remains challenging [28, 29, 30, 31, 32, 33].

Typically, electrodeposition of MnO_2_ involves electrolytes such as potassium permanganate (KMnO_4_) [34, 35], manganese acetate (Mn(CH_3_COO)_2_) [36], and manganese sulfate (MnSO_4_) [37], each offering distinct advantages and limitations. For instance, strong oxidizers like KMnO_4_‐based systems spontaneously redox deposit with minimal activation but often suffer from uncontrollable film growth, poor adhesion, and environmental concerns due to the nature of the strong oxidizing effects of the precursor [38]. Electrodeposition studies using MnSO_4_ precursors have demonstrated promising results, and MnSO_4_ in combination with H_2_SO_4_ results in a highly tunable and cost‐effective deposition solution, promoting uniform Mn^2^^+^ oxidation [39]; sulfate ions also suppress side reactions and assist in the formation of pure MnO_2_ phases [40]. Carbon cloth substrates provide good mechanical adhesion with active materials, which improves conductivity, accelerates charge transport, and results in decreased resistance [41]. Moreover, it has been shown that adjusting the deposition potential could tailor MnO_2_ crystallinity and phase composition [42].

Magneto‐electrodeposition of MnO_2_ can significantly influence the deposition process through magnetohydrodynamic (MHD) effects. The Lorentz force, generated by the interaction between ionic currents and applied magnetic fields, enhances convective flow in the electrolyte, leading to homogeneous ion distributions, increased mass transport, and denser film formation [43]. Magnetic‐field‐assisted synthesis has previously been applied in other electrochemical systems, such as in the growth of nano films [12] and conductive polymers [44], demonstrating notable improvements in morphology, crystallinity, and electrochemical performance. In energy storage applications, a few pioneering studies have explored the influence of magnetic fields. Wang et al. reported the preparation of magnetic‐field‐assisted synthesis polyaniline films for supercapacitors, showing superior electrochemical performance [45]. Our approach is distinct from traditional KMnO_4_‐based electrodeposition by employing a safer, environmentally benign electrolyte system and introducing a novel MED‐driven synthesis route [27, 46]. This work introduces a MED strategy that couples low‐field magnetic control during both the cathode synthesis and battery operation to regulate ion transport and cathode structure in Zn‐MnO_2_ systems. Unlike prior studies that focus on either magnetic field‐assisted depositions or high‐field electrochemical effects independently, this study demonstrates that modest magnetic fields can be used synergistically to enhance Mn^2+^ deposition kinetics, stabilize δ‐MnO_2_ formation, and dynamically modulate Zn^2+^ transport during cycling. This multi‐stage magnetic field integration establishes a scalable and energy‐efficient approach for controlling both material synthesis and electrochemical behavior in aqueous batteries.

## 2 Materials and Methods

All chemicals were used as received from suppliers without further purification unless otherwise specified. Sulfuric acid (3M H_2_SO_4_) and manganese sulfate monohydrate (1M MnSO_4_·H_2_O) were mixed to prepare an aqueous MnO_2_ precursor solution for MnO_2_ deposition onto commercially sourced carbon cloth from Fuel Cell. Prior to electrodeposition, the 7/16‐inch carbon cloth discs were pre‐soaked in the aqueous MnO_2_ precursor solution for 5 min to ensure thorough wetting. A 3D‐printed well‐cell configuration was employed, as shown in Figure S1, for MnO_2_ ED and MED, enabling the fabrication of MnO_2_ cathodes. A three‐electrode setup consisting of a platinum counter electrode, an Ag/AgCl reference electrode, and a copper lead was used for ED and MED. The pre‐soaked carbon cloth was placed on a titanium (Ti) disc, supporting and preventing solution leakage during ED and MED. For MED MnO_2_ cathode fabrication, the same protocol of ED was followed, and a block neodymium magnet (∼0.4T) was carefully positioned beneath the well‐cell during electrodeposition to study the effect of magnetic fields on MnO_2_ growth and electrochemical behavior. ED and MED were carried out at potentials ranging from 1.4 to 1.7 V for 20 min, unless otherwise stated. By comparing their electrochemical performance and charge transfer resistance (R_ct_), further optimization was performed by electrodepositing MnO_2_ at 1.5 V for 2, 5, 10, 20, and 30 min to determine the optimal deposition times for electrochemical performance evaluation. Zinc sulfate heptahydrate (ZnSO_4_·7H_2_O) was obtained from Amresco. 7/16‐inch diameter carbon cloth discs used for both ED and MED, glass fiber separators (Whatman, 24.0 cm circles, 180 µm thickness, 11 µm particle retention) were cut into 1/2‐inch diameter discs. Half‐inch Swagelok cells and fittings were sourced from Swagelok, while 1/2‐inch stainless steel rods and 0.85 mm thick titanium spacers were procured from McMaster‐Carr.

### 2.1 Fabrication and Electrochemical Testing of Batteries With Internal Magnets

To fabricate a half‐cell for electrochemical evaluation, electrodeposited and optimized free‐standing MnO_2_ cathodes were used. The free‐standing electrodeposited cathodes were tapped on a clean paper towel to remove excess electrolyte and then used for the fabrication of the corresponding cell. During battery assembly, 40 µL of 2 m ZnSO_4_ was added to the glass fiber separator to ensure electrolyte distribution. Zinc foil (0.2 mm thick) was used as the anode, cut into a half‐inch diameter disc. The assembled cells were left to rest for 24 h at open‐circuit voltage (OCV) to stabilize before further electrochemical testing. Disk magnets purchased from K&J Magnetics (1 mm thickness and ½ diameter) were incorporated into cells to test magnetic field effects during battery operation. Electrochemical characterization, including cyclic voltammetry (CV), galvanostatic charge/discharge (GCD) cycling, and electrochemical impedance spectroscopy (EIS), were performed using a Bio‐Logic BCS‐805 battery tester and PalmSens4 potentiostat (PalmSens B.V., Netherlands). Magnetic field strength at the electrode surface was measured using a Gauss meter (LATNEX MF‐30K).

### 2.2 Materials Characterization

Scanning electron microscopy (SEM) and energy‐dispersive X–ray (EDS) analysis were performed on anodes and cathodes on an FEI Apreo Volumescope. Fourier‐transform infrared spectroscopy (FTIR) was run on a Thermo Scientific Nicolet iS10 IR instrument. X–ray diffraction (XRD) was performed on electrodeposited cathodes using a Bruker D2 Phaser Powder X–ray Diffractometer. BRUKER TXRF S4‐TStar instrument is used to estimate Mn content in their corresponding MnO_2_ cathodes from ED and MED. To quantify Mn content in ED and MED MnO_2_ cathode samples using TXRF, the following protocol was followed: 10 µL of each sample (prepared in 90 µg/mL concentration) was mixed with 10 µg/mL Gallium (Ga) [90:10] internal standard in an Eppendorf tube and vortexed thoroughly. A 10 µL aliquot of the resulting solution was drop‐cast onto a pre‐cleaned quartz disc and dried on a hot plate under a fume hood. The prepared discs were then mounted onto T‐star TXRF trays and analyzed to determine Mn mass content. Transmission electron microscopy (TEM) and TEM diffraction were evaluated by using a Tecnai G2 TEM (2011) FEI Company (Field Emission Instruments), and Electron Paramagnetic Resonance (EPR) was measured by using ELEXSYS‐II E500 CW‐EPR.

## 3 Results and Discussion

### 3.1 Magneto‐Electrodeposition (MED) Strategy and Reaction Mechanism

**FIGURE 1:**
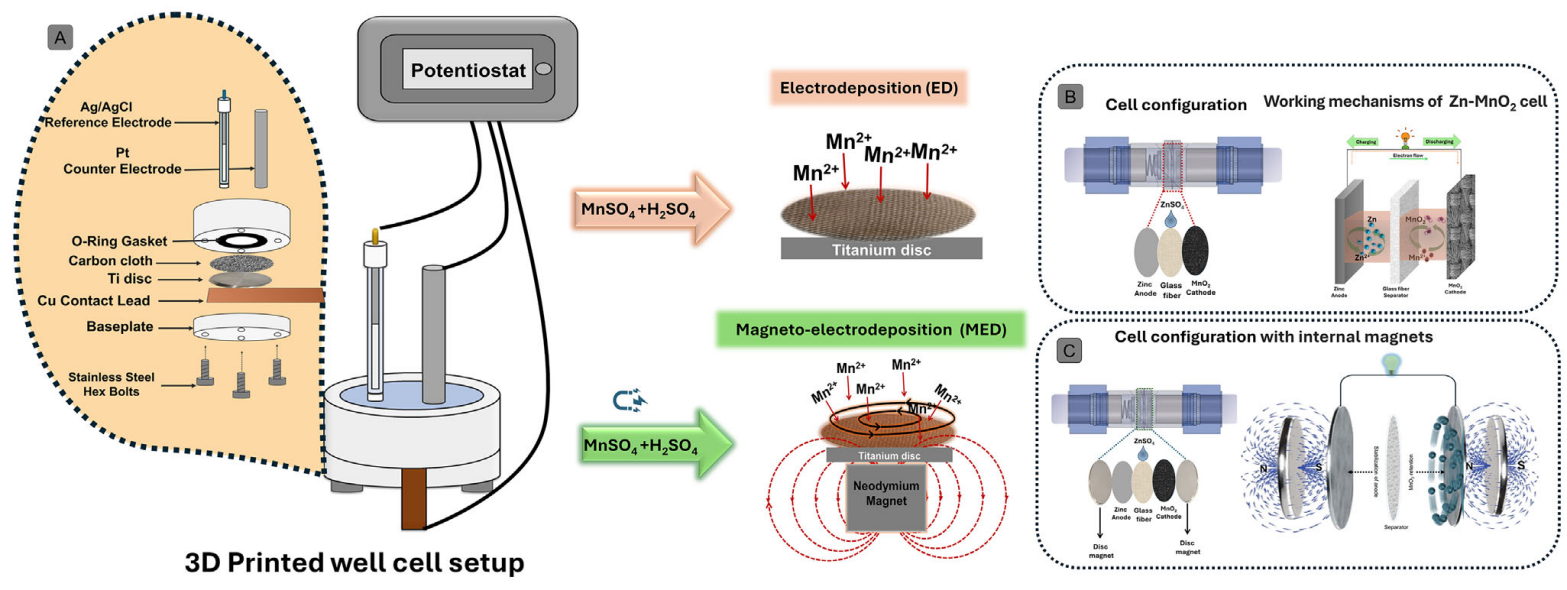
Schematic illustration of Lorentz‐force‐driven MHD ion transport in magnetically assisted Zn‐MnO_2_ batteries (a) comparison of ED and MED of MnO_2_ on carbon cloth under a ∼0.4T magnetic field using a custom 3D‐printed electrochemical cell for MnO_2_ cathode fabrication with corresponding *i–t* curves and cross‐sectional SEM images for morphological comparison, (b) Swagelok cell configuration and working mechanism of a conventional Zn‐MnO_2_ aqueous battery system, and (c) Magnetically assisted cell configuration employing internal magnets to enhance electrochemical performance.

Figure 1A illustrates a custom 3D‐printed well‐cell used for MnO_2_ electrodeposition on carbon cloth (Ti‐supported) in a three‐electrode configuration, with carbon cloth (Cu lead) as the working electrode, Pt as the counter electrode, and Ag/AgCl as the reference electrode. MED was performed under identical conditions as ED, with an external neodymium magnet (∼0.4 T), to investigate the effects of magnetic fields on ion transport and film growth (Figure S1). MnO_2_ was deposited from a 1 M MnSO_4_‐3M H_2_SO_4_ electrolyte at voltages ranging from 1.4–1.7 V for 20 min. The deposition voltage range of 1.4–1.7 V was selected as a screening window to evaluate the effect of oxidative driving force on MnO_2_ nucleation and growth. 1.7 V serves as an upper‐bound condition where faster Mn^2+^ oxidation can increase deposition rates but may also promote parasitic reactions, local pH perturbation, oxygen evolution, and formation of less favorable MnO_2_ polymorphs.

$$Mn^{2+}:\;Mn^{2+}+2H_{2}O\;\to \;\mathrm{Mn}O_{2}+4H^{+}+2e^{-}$$

In ED, ion transport is governed by diffusion and migration, leading to concentration gradients, non‐uniform nucleation, and porous MnO_2_ growth with limited δ‐phase ordering. In contrast, MED induces Lorentz‐force‐driven MHD convection (Equation 1).

(1) F=J×B

MHD convection reduces the effective diffusion layer thickness, enhances Mn^2^^+^ mass transport, and promotes uniform nucleation and dense MnO_2_ deposition. The *i–t* curves in Figure S2 confirm higher deposition currents for MED at increased potentials, indicating accelerated Mn^2^^+^ oxidation kinetics. Figure 1B shows the conventional Zn‐MnO_2_ cell, where Zn oxidation (Zn → Zn^2^^+^ + 2e^−^) is coupled with MnO_2_ redox/intercalation (MnO_2_+ Zn^2^^+^ + 2e^−^ ↔ ZnMnO_2_). However, performance is limited by Zn^2^^+^ diffusion resistance, concentration polarization, and Mn dissolution. To address these issues, Figure 1C presents a magnetically assisted cell design using external disc magnets placed on both sides of the cell. The resulting Lorentz force induces MHD convection (as shown in Video S1), enhancing electrolyte mixing and homogenizing ion concentration near electrodes, which is not observed in ED (Video S2).

### 3.2 Morphology and Elemental Distribution of MnO_2_ Cathodes

**FIGURE 2:**
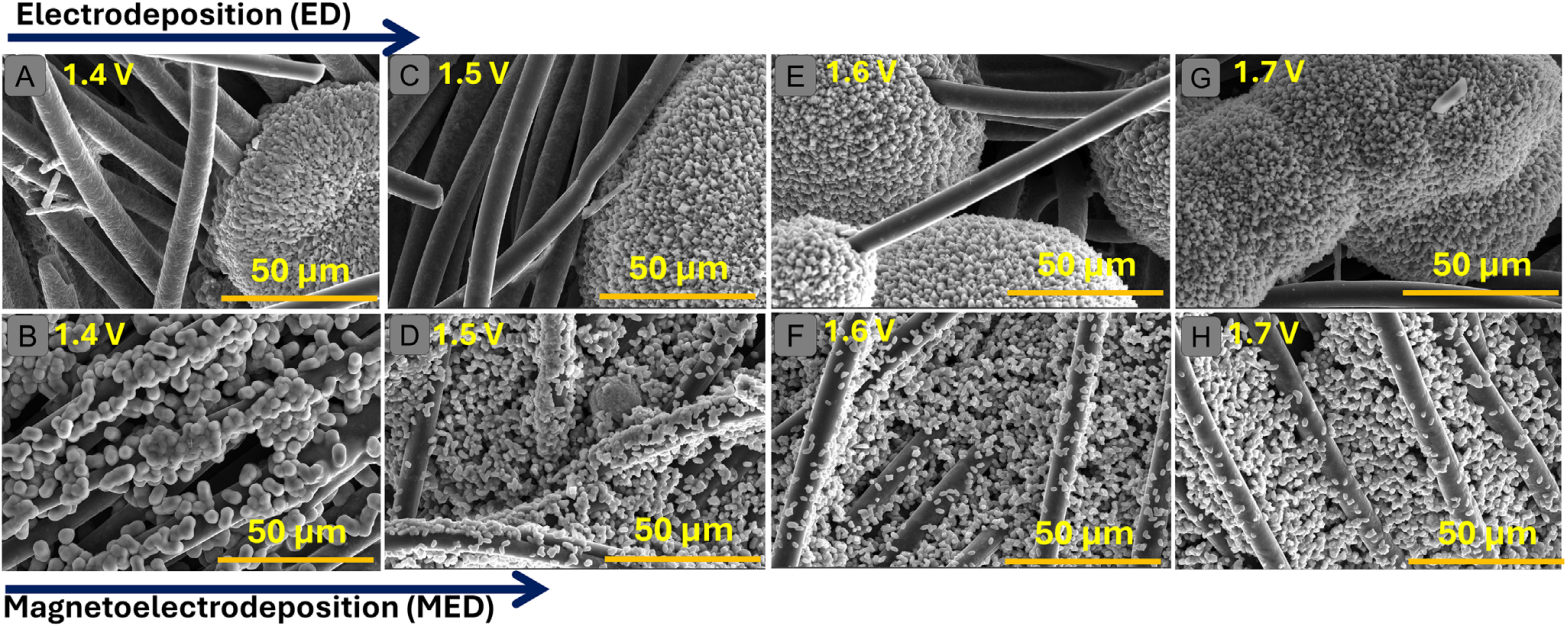
SEM images of ED and MED MnO_2_ on carbon cloth cathodes showing morphological differences between ED and MED at varying deposition voltages (1.4–1.7 V) for 20 min.

SEM images (Figure 2) reveal distinct differences in deposition density between ED and MED MnO_2_ cathodes. In the absence of a magnetic field, MnO_2_ deposition is governed by diffusion and migration, resulting in non‐uniform growth, random nucleation, and particle aggregation (Figure 2, top panels). Such heterogeneous morphology can adversely affect charge transfer and cycling stability due to limited contact between MnO_2_ particles. In contrast, MED produces significantly improved surface coverage with a dense and homogeneous MnO_2_ distribution (Figure 2, bottom panels). Particle aggregation is suppressed through local convection near the carbon‐cloth surface instigated by the MHD effect. Additional SEM images (Figures S3 and S4) further confirm the uniform coating of MnO_2_ on carbon fibers under magnetic‐field‐assisted conditions compared to pristine substrates. Elemental mapping via EDX (Figures S5 and S6) demonstrates a more uniform Mn distribution in MED samples, along with increased Mn loading. At 1.5 V, the Mn content increases from ∼21.6 wt.% (ED) to ∼26.6 wt.% (MED), corresponding to ∼22% enhancement (Table S1). This improvement highlights the effectiveness of MHD convection in enabling denser and more homogeneous MnO_2_ deposition. Importantly, MED cannot be described simply as bulk electrolyte agitation. Mechanical stirring reduces macroscopic concentration gradients, whereas Lorentz‐force‐driven MHD convection is generated locally by the interaction between ionic current density and the applied magnetic field. Because the Lorentz force scales with J × B, the resulting flow is coupled to electrochemically active regions of the electrode surface. This localized convection decreases the effective diffusion layer thickness, refreshes Mn^2+^ near the carbon‐cloth interface, reduces spatial variations in Mn^2+^ depletion and proton accumulation, and promotes more uniform MnO_2_ nucleation. These effects explain the denser and more homogenous MnO_2_ coatings observed for MED relative to conventional ED.

### 3.3 Structural and Compositional Analysis

**FIGURE 3:**
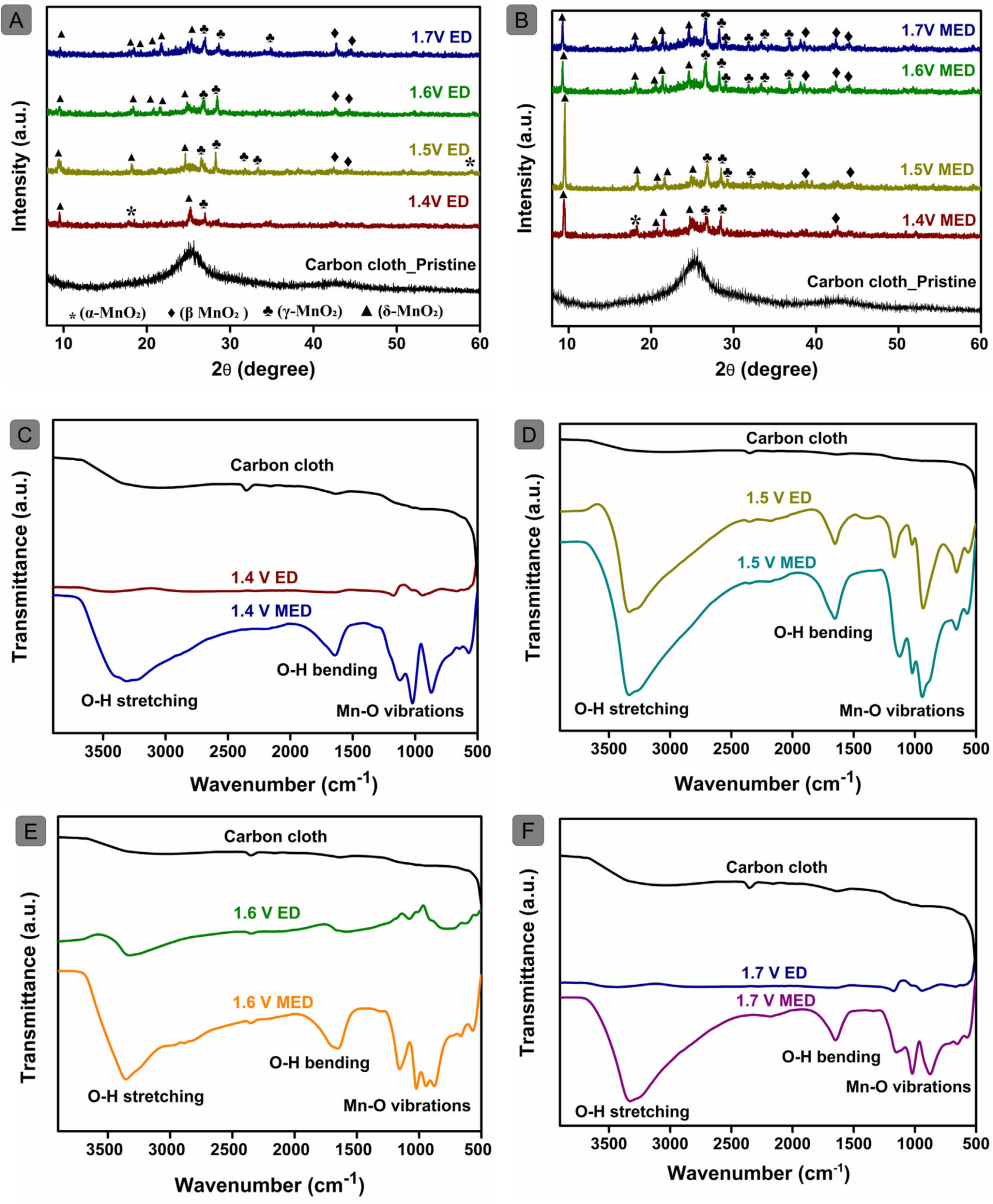
(a) XRD analysis showing phase evolution of MnO_2_ cathodes electrodeposited at different deposition voltages (1.4–1.7 V) through ED and MED (b) and FTIR spectra of ED and MED MnO_2_ cathodes at different voltages: (c) 1.4 V, (d) 1.5 V, (e) 1.6 V, and (f) 1.7 V (c–f). Standard reference patterns and crystallographic information are provided in Figure S7.

To provide insights into the role of applied magnetic fields on the resulting phase evolution and crystallinity of the resultant MnO_2_ cathodes, XRD and FTIR characterizations were performed. Figure 3a,b reveals the significant impact of magnetic field assistance on the crystallization and phase evolution of MnO_2_ cathodes. Across the voltage range of 1.4–1.7 V (applied for 20 min), all the MnO_2_ cathodes consistently display phase diversity; however, MED can be seen to profoundly influence both phase purity and crystallinity. Using the 1 M MnSO_4_·H_2_O and 3 M H_2_SO_4_ electrolyte system, ED at 1.4 and 1.5 V predominantly yields δ‐MnO_2_, confirmed by strong reflections at 9.59° (001), 17.9° (001), and 22.37° (100), characteristic of the layered birnessite‐type structure [47]. However, these peaks are relatively broad and less intense, indicating lower crystallinity and more disordered growth. As voltage increases (1.6–1.7 V), the XRD patterns shift toward tunnel‐structured phases, evidenced by γ‐MnO_2_ peaks at 27.82° (110) and β‐MnO_2_ peaks at 34.19°, 39°, and 43.5° (110, 211, 301), alongside α‐MnO_2_ at 46.2° (020). This trend shows that higher potentials inherently favor the formation of more crystalline, dense phases, but at the cost of layered δ‐MnO_2_. It is established that δ‐MnO_2_ is especially favorable for aqueous Zn^2^^+^ intercalation due to its open layered structure, and β‐MnO_2_, though highly crystalline, limits Zn^2^^+^ intercalation due to its tight tunnel dimensions [48]. Both γ‐MnO_2_ and α‐MnO_2_ possess tunnel‐type crystal structures that facilitate Zn^2^^+^ ion transport, while offering good structural stability, making them highly suitable for prolonged cycling in aqueous Zn‐ion batteries. Importantly, the δ‐MnO_2_ peak at 9.59° intensifies under magnetic fields, particularly at 1.5 V, suggesting that magnetic fields not only boost crystallinity but also stabilize the layered phase, and low‐angle reflections at 20.76° and 21.54° appear exclusively in MED MnO_2_ cathode samples, pointing to either expanded interlayer spacing or the formation of novel hydrated δ‐MnO_2_ polymorphs [49, 50, 51]. Such phases are absent using the conventional ED protocol, reinforcing the unique role of magnetic effects in tailoring crystal architecture.

The peak at 9.59°, which corresponds to basal spacing (the distance between atomic planes that give rise to diffraction peaks) of ∼9.2–9.8 Å, highlights a slight voltage‐dependent crystallization pathway at 1.4 V, where insufficient oxidation leads to weakly defined δ‐MnO_2_. The applied potential of 1.5 V emerges as optimal for δ‐MnO_2_, balancing Mn^2^^+^ oxidation kinetics and suppressing side reactions that occur at 1.7 V, where overoxidation and increased driving force shift phase equilibrium toward β‐phase and γ‐phases, reducing δ‐MnO_2_ content. At 1.6 and 1.7 V, overoxidation may occur and promote transformation to more crystalline, but non‐layered MnO_2_ phases like γ or β‐MnO_2_. This can lead to decomposition of δ‐MnO_2_ or formation of Mn_2_O_3_/Mn_3_O_4_ side phases, reducing the relative intensity of the 9.59° peak. Figure S8 presents crystallographic models of MnO_2_ polymorphs, and (Table S2) lists crystal systems and structural characteristics of MnO_2_ polymorphs (Figure S8) from the literature for reference. MED mitigates phase drift, preserving δ‐MnO_2_ integrity and advancing multi‐phase coexistence with enhanced crystallinity across the voltage spectrum. The preferential formation of δ‐MnO_2_ under MED is attributed to the combined effects of moderated concentration gradients, local pH stabilization, and hydration‐controlled crystal growth. Oxidation of Mn^2+^ to MnO_2_ generates protons at the electrode surface, and under conventional ED, local Mn^2+^ depletion and proton accumulation can produce heterogeneous nucleation environments. Lorentz‐force‐driven MHD convection decreases the effective diffusion layer thickness and continuously refreshes Mn^2+^ near the carbon‐cloth interface, producing a more uniform interfacial environment. At the optimized 1.5 V condition, this moderated and hydrated growth environment favors layered birnessite‐like δ‐MnO_2_. At higher potentials, the increased oxidative driving force can promote more compact or mixed tunnel‐type phases, reducing the relative selectivity toward δ‐MnO_2_. Additionally, MED amplifies peak sharpness and intensity for β, γ, and α phases as well, particularly at higher voltages, indicating that its benefits extend beyond δ‐MnO_2_ stabilization to overall phase densification and order. This results in compact films with superior mechanical robustness, crucial for cycling stability. In contrast, ED cathodes exhibit broader peaks, pointing to more porous, less ordered structures prone to capacity fade and mechanical degradation.

FTIR spectra (Figure 3c–f) further support these findings; all samples exhibit characteristic Mn─O and Mn─O─Mn vibrations, confirming MnO_2_ formation. Compared to ED, MED samples show sharper and more intense Mn–O bands, indicating improved crystallinity and bonding. Enhanced O–H features suggest increased surface hydroxylation, which can facilitate proton‐assisted charge storage, while more pronounced sulfate‐related bands indicate electrolyte incorporation that may contribute to pseudocapacitive behavior. In the low‐voltage (1.4 V) electrodeposition, peaks at 1176, 941, and 659 cm^−^^1^ are weak but suggest Mn─O vibrations typical of MnO_2_ frameworks. However, under the influence magnetic fields at 1.4 V, additional peaks appear at 3309 cm^−^^1^ (O─H stretching of adsorbed water), 1635 cm^−^^1^ (O─H bending), and more prominent Mn─O related vibrations at 1141, 1029, 875, and 578 cm^−^^1^, indicating enhanced hydroxylation and more developed oxide network formation, likely facilitated by magnetic‐field‐induced convection that promotes more uniform nucleation. At 1.5 V, both ED and MED MnO_2_ cathodes exhibited strong O─H stretching (∼3328 cm^−^^1^) and bending (∼1666 cm^−^^1^), but MED cathodes also show sharper Mn‐O peaks at 1137, 1014, and 937 cm^−^^1^, as well as lower‐frequency bands at 655 and 563 cm^−^^1^, signifying denser MnO_2_ frameworks. The enhanced peak clarity and intensity for MnO_2_ fabricated in the presence of magnetic fields provide further evidence that Lorentz‐force‐driven MHD effects improve deposition uniformity and crystallinity. At 1.6 V, ED samples present a broad O–H peak at 3343 cm^−^^1^ and features at 1500 and 782 cm^−^^1^ (broad), reflecting less‐defined oxide bonding likely due to rapid deposition and poor organization. In contrast, MED MnO_2_ cathodes exhibit more resolved peaks at 3374, 1639, 1149, 1025, and 937 cm^−^^1^, with small peaks at 875, 655, and 582 cm^−^^1^, indicating a more crystalline MnO_2_ structure. The magnetic field likely mitigates the disordered deposition caused by high current density via MHD stabilization of ion flow. At 1.7 V, ED cathodes show only weak Mn–O peaks (1184, 952, 678 cm^−^^1^), suggesting poor coverage of MnO_2_ across cathodes. However, MED cathodes feature strong O–H and Mn–O signals (3320, 2183, 1658, 1141, 1025, 607, 671 cm^−^^1^), pointing to a well‐developed oxide lattice. The strong 2183 cm^−^^1^ band could be associated with surface‐bound species or residual sulfate complexes stabilized within the MED deposition.

### 3.4 Charge Transport and Interfacial Properties

**FIGURE 4:**
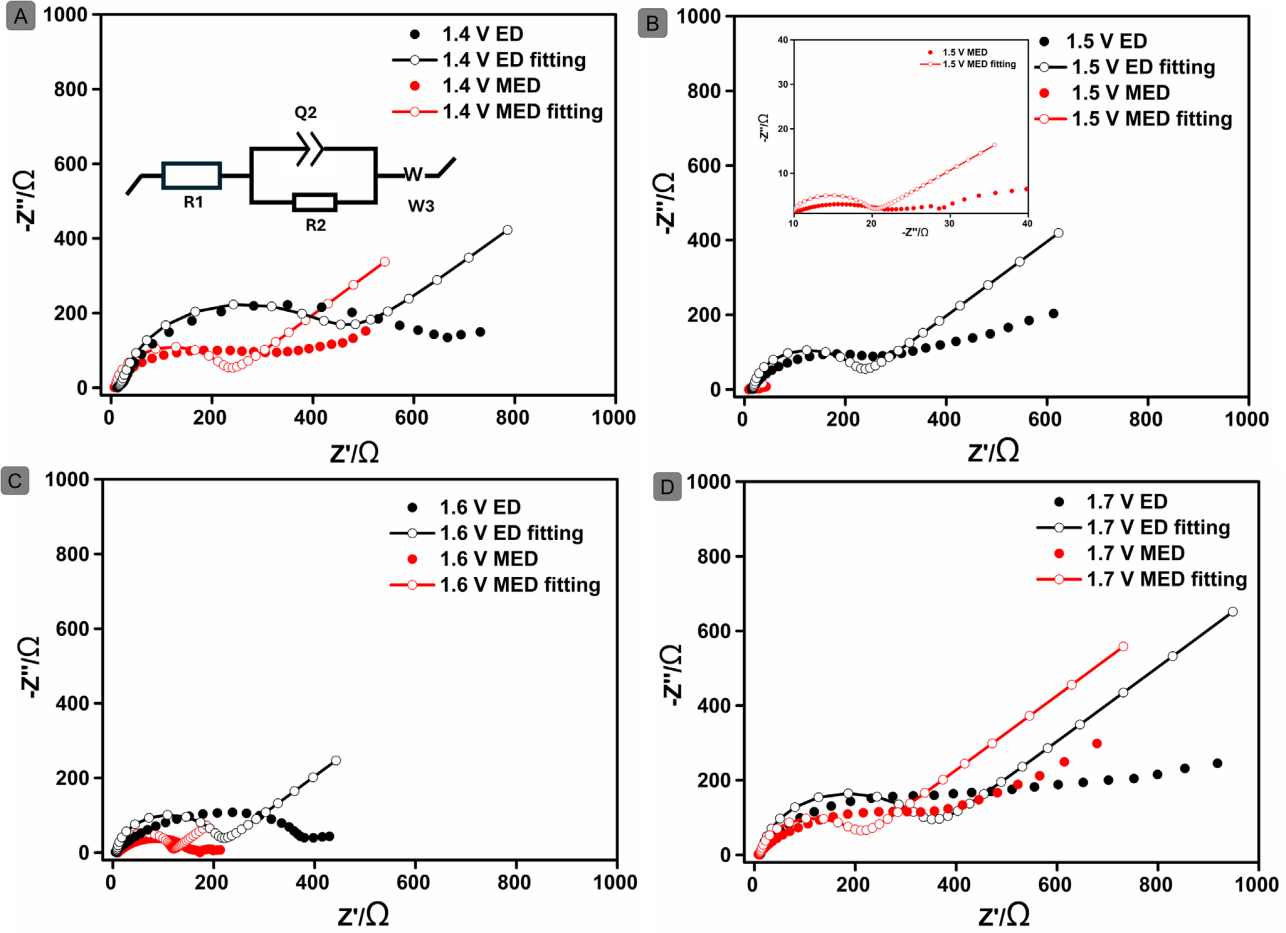
(a–d) Nyquist plots of EIS measurements of electrodeposited and MED MnO_2_ cathodes at different deposition voltages (1.4–1.7 V) measured in a fully 3D printed well‐cell in 1 M ZnSO_4_ at OCV from 10^5^ to 0.1 Hz.

Nyquist plots (Figure 4) reveal significantly improved charge‐transfer kinetics for MED MnO_2_ cathodes compared to conventional ED cathodes. Across all deposition potentials (1.4–1.7 V), MED consistently exhibits lower charge‐transfer resistance (R_ct_), indicating enhanced interfacial charge transport (Table S3). The most pronounced improvement is observed at 1.5 V, where R_ct_ decreases from 187.7 Ω (ED) to 9.7 Ω (MED), corresponding to a 94% reduction. At different potentials, MED cathodes delivered substantial decreases in R_ct_, confirming the general effectiveness of MED in improving electrochemical kinetics, relevant fitting information for ED and MED at 1.5 V is mentioned in Table S4. This enhancement is attributed to the formation of dense, uniform, and well‐adhered MnO_2_ films with improved electrical connectivity and ion diffusion pathways. The superior charge transport properties of MED cathodes are consistent with the morphological and structural results (Figures 2 and 3). Of these potentials, 1.5 V emerges as the optimal deposition condition, balancing efficient Mn^2^^+^ oxidation with controlled film growth, resulting in minimal interfacial resistance and favorable kinetics. Cyclic voltammetry (Figure S9) further supports these findings. ED cathodes exhibit broad and poorly defined redox peaks, indicating sluggish kinetics and limited reversibility. In contrast, MED cathodes display sharper and more distinct redox peaks with higher current response, reflecting improved electrochemical reversibility and faster reaction kinetics. This confirms that MED facilitates efficient ion/electron transport. To further quantify the influence of magnetic fields‐MHD effect on ion transport kinetics, cyclic voltammetry was performed at various scan rates and apparent transport coefficients were estimated using the Randles–Ševčík equation (Figure S10). Cyclic voltammograms were obtained in the presence and absence of 0.4 T magnetic fields using pristine carbon cloth as the working electrode in the original deposition solution (3 M H_2_SO_4_ and 1 M MnSO_4_). In the presence of a magnetic field, the observed slope (i_p_‐v^1/2^) was steeper than the slope obtained in the absence of a magnetic field, corresponding to an increased apparent Mn^2+^ transport coefficient 1.07 × 10^−8^cm^2^ s^−^^1^ vs. 7.61 × 10^−9^ cm^2^ s^−^^1^. This result is consistent with EIS analysis, where MED decreases charge‐transfer resistance relative to ED, confirming improved interfacial charge transfer and ion‐accessibility.

### 3.5 Cross‐sectional Morphology and Elemental Analysis of MnO_2_ Cathodes

**FIGURE 5:**
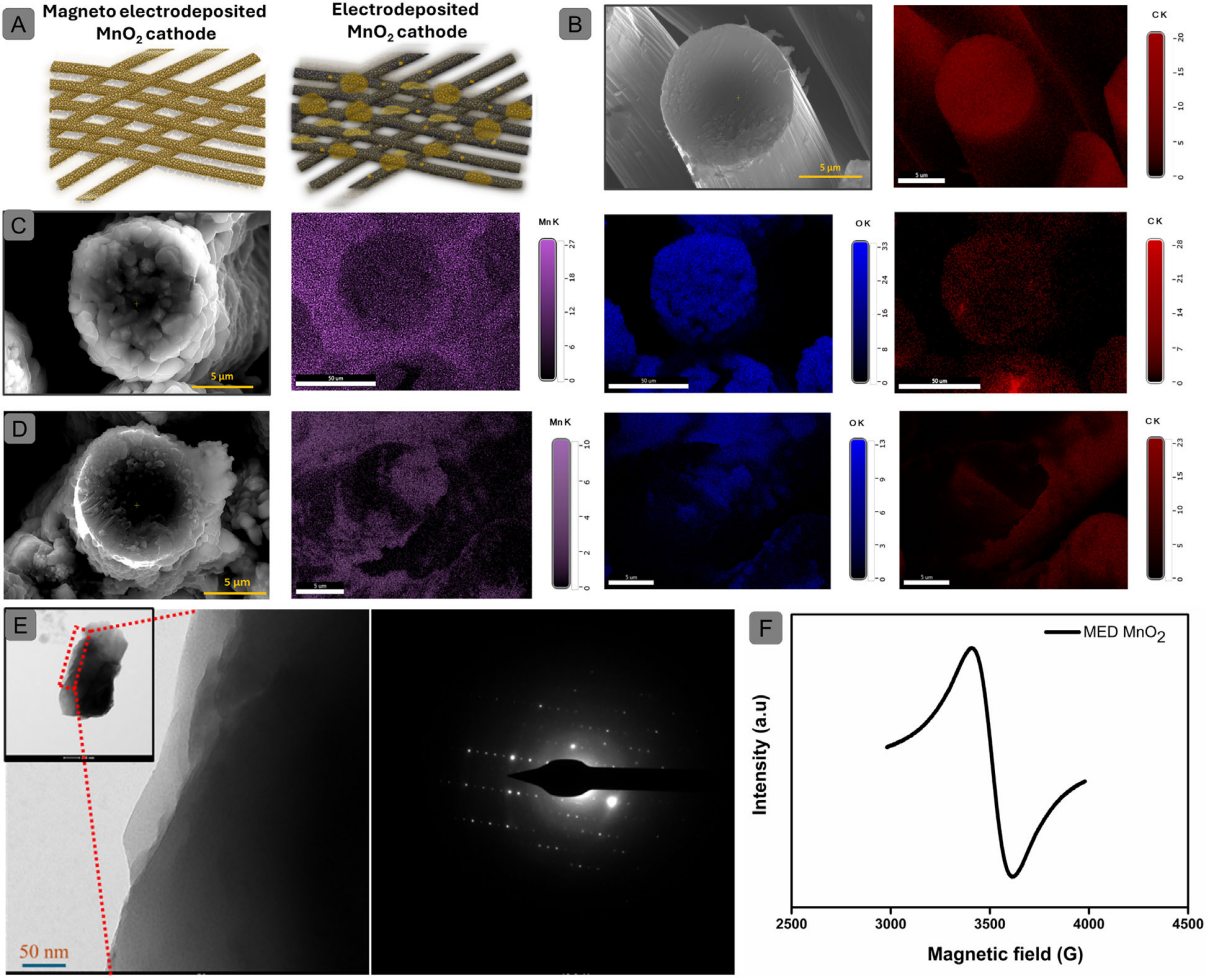
Cross‐sectional SEM image and EDS of individual fibers: (a) graphical representation of MED vs ED MnO_2_ cathode, (b) Pristine carbon cloth fiber. Cross‐sectional SEM and its corresponding EDS mapping results, (c) MED cathode, and (d) ED cathode at 1.5 V for 20 min, (e) microstructural TEM and Selected Area Electron Diffraction (SAED) analysis and (f) its corresponding EPR spectrum confirming Mn^4^^+^ centers of magnetic characterization of MED‐MnO_2_.

Cross‐sectional SEM analysis (Figure 5) reveals significant differences in MnO_2_ deposition between ED and MED cathodes. The pristine carbon fiber exhibits a diameter of ∼9–10 µm (Figure 5b), whereas MED results in a coating with an increased fiber diameter of ∼11–11.5 µm, corresponding to a uniform layer thickness of ∼1–1.5 µm (Figure 5c). In contrast, ED samples show discontinuous and non‐uniform MnO_2_ coverage (Figure 5d), consistent with surface SEM observations (Figure 2). EDS mapping further confirms a homogeneous distribution of Mn and O across the MED‐coated fibers, while ED samples exhibit irregular and sparse Mn signals. Quantitative analysis shows a substantial increase in Mn content for MED cathodes, with ∼22% higher surface Mn (EDX) and ∼14.6% higher total Mn loading (TXRF) (Figure S11). This confirms more efficient and denser MnO_2_ deposition under the MED protocol.

TEM analysis (Figure 5e) reveals a nanosheet‐like layered morphology characteristic of δ‐MnO_2_ (birnessite), consisting of stacked lamellar structures. The corresponding SAED pattern displays polycrystalline ring features dominated by δ‐MnO_2_ reflections, with minor contributions from secondary phases. EPR spectra (Figure 5f) show a broad resonance with g ≈ 2.02, confirming the presence of Mn^4^^+^ in a strongly coupled lattice and the absence of isolated Mn^2^^+^ species [52, 53] and the calculation of the g‐factor is provided in Supporting Information. Together with XRD results, these findings confirm that δ‐MnO_2_ is the dominant phase in MED cathodes, with minor secondary phases that do not significantly affect the overall structure. The formation of uniform, compact coatings with enhanced Mn loading and controlled phase composition highlights the effectiveness of MED in tailoring MnO_2_ electrodes for improved electrochemical performance.

### 3.6 Electrochemical Performance of ED vs MED Cathodes

To evaluate the electrochemical performance of the fabricated MnO_2_ cathodes, we assembled Swagelok‐type battery cells with Zn anodes and 2 M ZnSO_4_ electrolyte for battery operation. The relevant reactions of the Zn‐MnO_2_ battery during the charge process is as follows:

$$\begin{array}{cc} \mathrm{Cathode}:\;\; & Mn^{2+}+2H_{2}O=\mathrm{Mn}O_{2}+4H^{+}+2e^{-},\;\;\\ \; & E^{\circ }=1.23\;V\;\;\mathrm{vs}.\;\mathrm{SHE} \end{array}$$

$$\mathrm{Anode}:\;\;Zn^{2+}+2e^{-}=\mathrm{Zn},\;\;E^{\circ }=-0.76\;V\;\;\mathrm{vs}.\;\mathrm{SHE}$$

$$\begin{array}{cc} \mathrm{Redox}:\;\; & Mn^{2+}+Zn^{2+}+2H_{2}O=\mathrm{Mn}O_{2}+4H^{+}+\mathrm{Zn},\;\\ \; & E^{\circ }=1.99\;V\;\;\mathrm{vs}.\;\mathrm{Zn}/Zn^{2+} \end{array}$$

**FIGURE 6:**
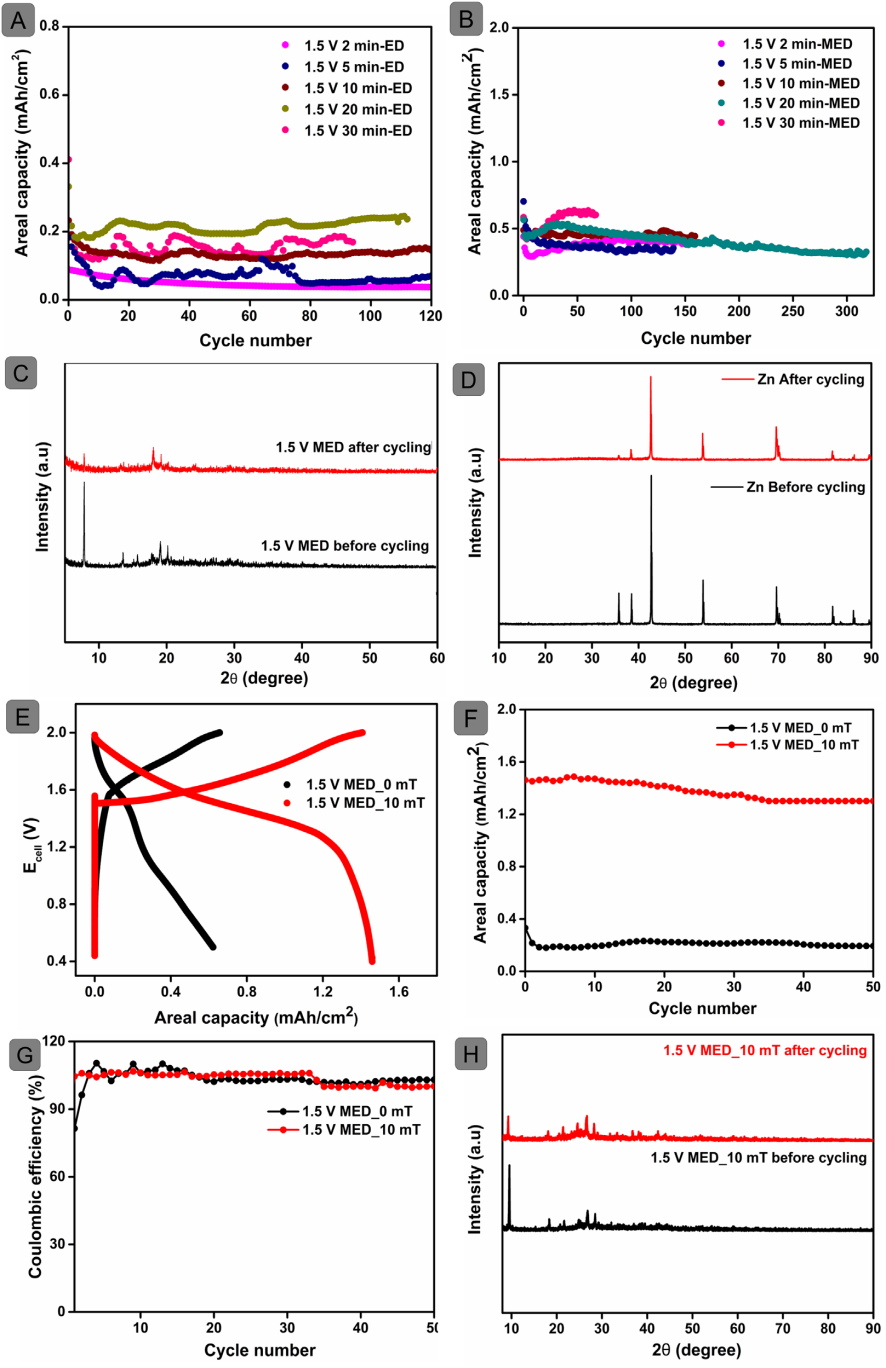
Electrochemical performance of MED MnO_2_ cathodes with and without internal magnets: (a) GCD cycling performance of full Zn‐MnO_2_ cell incorporating ED MnO_2_ cathodes fabricated at 1.5 V for different times at 1.2 C, (b) GCD cycling performance of full Zn‐MnO_2_ cell incorporating MED MnO_2_ cathodes at 1.5 V for different times at 1.2 C, (c,d) XRD patterns of 1.5 V MnO_2_ MED cathode and Zn anode before and after cycling, (e) GCD voltage profiles of full Zn‐MnO_2_ cell incorporating MED MnO_2_ cathodes operated with and without an applied internal magnetic field. (f) Cycling stability comparison of Zn‐MnO_2_ cells assembled with MED MnO_2_ cathodes under 0 and 10 mT magnetic field conditions, (g) Coulombic efficiency during long‐term cycling, and (h) XRD patterns of MED MnO_2_ cathodes before and after cycling. These plots are from the average of 3 cells.

Initial galvanostatic charge‐discharge (GCD) profiles (Figure S12) show that MED cathodes consistently deliver higher areal capacities than their ED counterparts across all deposition potentials studied (1.4–1.7 V). The most significant enhancement is observed at 1.5 V, where MED achieves higher initial areal capacity ∼0.62 mAh cm^−^^2^ compared to ∼0.40 mAh cm^−^^2^ for ED (∼55% improvement), as shown in (Figure S12). Based on combined structural (XRD), morphological (SEM/EDX), and kinetic (EIS) analyses, 1.5 V was selected for further optimization in the battery configuration. Both ED and MED were performed for different amounts of time (Figure 6a,b), revealing that a deposition duration of 20 min yields the best performance for MED cathodes and their corresponding Coulombic efficiencies (Figure S13). Compared to ED, the Zn‐MnO_2_ full cells composed of MED cathodes demonstrated significantly higher average capacity over 100 cycles, delivering 0.46 mAh cm^−^^2^ versus 0.24 mAh cm^−^^2^ with capacity retentions of ∼73% and 72%, respectively. Their corresponding charge‐transfer resistances from Nyquist plots and their redox behaviors are shown in Figure S14. Notably, the MED cathode maintains stable cycling for over 315 cycles (retaining 53% of its initial capacity), significantly outperforming the ED cathode, which fails after ∼130 cycles. Short deposition times (2–10 min) result in insufficient MnO_2_ loading and lower capacities, while prolonged deposition (30 min) produces higher initial capacity (∼0.65 mAh cm^−^^2^) but rapid degradation due to higher mass loading and polymorphism. These results highlight that 1.5 V for 20 min provides an optimal balance between active material loading, film uniformity, and structural stability. A direct comparison across deposition times further confirms the superiority of MED, with capacities ranging from ∼0.25–0.65 mAh cm^−^^2^, compared to ∼0.07–0.20 mAh cm^−^^2^ for ED. The initial Coulombic efficiency variability observed for some electrodes is mainly attributed to wetting/activation of the binder‐free MnO_2_/carbon‐cloth interface and the low absolute capacity of underloaded electrodes, especially for short deposition times. After the activation period, the optimized 1.5 V, 20 min MED cathode exhibits Coulombic efficiency approaching ∼100%, supporting reversible Zn‐ MnO_2_ cycling. XRD analysis of the MED MnO_2_ cathode (1.5 V, 20 min) before and after cycling (Figure 6c) shows no new phase formation, confirming the stability of MED cathodes. The characteristic peaks of δ‐, γ‐, and other MnO_2_ polymorphs are largely preserved after prolonged cycling, with only a slight decrease in δ‐MnO_2_ (001) intensity, indicating minor structural rearrangement during Zn^2^^+^ intercalation/deintercalation. This stability is attributed to the binder‐free architecture and strong adhesion achieved via the MED protocol. To probe the effect of MED vs. ED cathodes on ion transport, we performed cyclic voltammetry scan rate analysis on MED and ED cathodes in the Zn‐MnO_2_ battery electrolyte. Plots of i_p_ vs. v^1/2^ reveal a steeper slope for MED compared to ED cathodes, confirming enhanced Zn^2+^ ion transport in the system with calculated apparent transport coefficients of 1.53 × 10^−8^ and 1.06 × 10^−8^ cm^2^ s^−^^1^ for MED and ED, respectively. These results are consistent with the lower charge‐transfer resistance values obtained in the MED cells. Because these values reflect the combined influence of morphology, wetting, porosity, and redox accessibility, they are described as apparent electrode‐level transport coefficients rather than intrinsic electrolyte diffusion coefficients.

### 3.7 Effect of Internal Magnetic Field on Battery Performance

To further enhance performance, internal magnetic fields were applied during battery operation. Among these, 10 mT delivered the best performance, providing an optimal balance between enhanced ion transport and system stability. The magnetic fields used during cathode fabrication and full‐cell operation serve different purposes and should not be directly compared as equivalent transport conditions. During MED, the ∼0.4 T magnetic field is applied to a small volume well‐cell under electrodeposition current, where MHD convection directly affects Mn^2+^ supply and MnO_2_ nucleation. During full‐cell cycling, the internal magnets generate a lower magnetic field of ∼10 mT at the electrode surface in a confined Swagelok geometry. MED controls cathode fabrication, whereas internal magnetic fields modulate battery operation. These two configurations produce related but distinct transport environments. Voltage profiles (Figure 6e) show a more stable discharge plateau under magnetic‐field‐assisted operation, indicating reduced polarization and improved reaction kinetics. The cycling performance of MED cathodes (Figure 6f) demonstrates a substantial increase in areal capacity under a 10 mT magnetic field (1.32 mAh cm^−^^2^ after 50 cycles) compared to the 0 mT system (0.33 mAh cm^−^^2^ after 50 cycles). Importantly, both systems employ identical MED cathodes, confirming that the observed performance enhancement arises solely from the applied magnetic field during battery operation. The Coulombic efficiency remains high (∼100%) in both cases, indicating good reversibility (Figure 6g). However, this improvement is accompanied by reduced cycling stability. With no magnetic field applied, MED cathodes (MED 0 mT) maintained stable operation for over 300 cycles, whereas cells incorporating internal magnets exhibited faster capacity fading, with significant decay after ∼50 cycles (10 mT). This behavior highlights a capacity‐stability trade‐off: enhanced ion transport under magnetic‐field‐induced convection improves the utilization of active MnO_2_ sites but may accelerate degradation processes such as Mn dissolution and structural distortion. Additionally, magnetic‐field‐induced convection may promote electrolyte utilization along with the MnO_2_ cathode, increasing the fraction of electrochemically accessible active material for the redox process. Because full‐cell operation involves both the MnO_2_ cathode and Zn anode, the effect of the internal magnetic field cannot be assigned solely to cathode‐side transport. Post‐cycling SEM analysis of Zn anodes recovered from cells operated at 0 and 10 mT reveals that magnetic‐field‐assisted cycling has the beneficial effect of smoothing the Zn surface, which has been previously reported [54, 55]. Compared to the 0 mT cell, Zn electrodes cycled under magnetic fields show decreased surface roughness (Figure S16). Therefore, the enhanced capacity observed under 10 mT is attributed to improved short‐term active material utilization and reduced polarization, whereas the accelerated capacity fading is attributed largely to degradation at the MnO_2_ cathode.

To further evaluate the effect of magnetic field strength, MED cathodes were tested under fields ranging from 10 to 30 mT (Figure S17). Initial discharge capacities increased with increasing field strength, with 10 mT delivering 1.46 mAh cm^−^^2^, 20 mT achieving 1.63 mAh cm^−^^2^, and 30 mT reaching 1.76 mAh cm^−^^2^. However, this increase in initial capacity occurs at the expense of cycling stability, as higher magnetic field strengths lead to earlier capacity fading compared to the 10 mT condition. Post‐cycling XRD analysis (Figure 6h) confirms that the overall phase structure is retained under magnetic‐field‐assisted operation, indicating that the observed performance degradation is primarily kinetic rather than structural. Overall, internal magnetic fields enhance capacity by improving ion transport and reducing polarization, with 10 mT identified as an optimal condition balancing performance and stability. Tables S5 and S6 compares MED MnO_2_ cathodes with recently reported aqueous Zn‐MnO_2_ batteries. We note that the cycling life of the present binder‐free MED MnO_2_ cathode is shorter than some highly engineered MnO_2_ composite systems reported in the literature. However, the goal of this work is not to claim record cycling performance, but to demonstrate that magnetic‐field‐assisted electrodeposition can directly improve the morphology, phase composition, Mn loading, charge‐transfer resistance, and areal capacity of electrodeposited MnO_2_. Compared with conventional ED, MED provides a simple room‐temperature fabrication route that does not require binders, high‐temperature treatment, or complex additives. To assess the influence of counter electrode configuration on the MED process, MnO_2_ cathodes were deposited using the optimized 1.5 V for 20 min protocol with a large‐surface‐area carbon cloth counter electrode. The chronoamperometric response (Figure S18a) shows an initially higher current followed by gradual decay, characteristic of nucleation and growth during MnO_2_ electrodeposition. The slightly elevated current compared to earlier depositions is attributed to improved charge balance and reduced polarization enabled by the larger counter electrode area, facilitating more efficient charge compensation and stable ion transport.

The electrochemical performance of the resulting MED MnO_2_ cathode (Figure S18b) exhibits a stable self‐discharge profile with a distinct plateau at ∼1.6–1.7 V, indicating reversible Zn^2^^+^ insertion/extraction within the MnO_2_ structure. Nyquist plots (Figure S18c) reveal a reduction in charge‐transfer resistance after cycling, suggesting improved electrode wetting and activation of electrochemically accessible sites. Furthermore, the cycling performance (Figure S18d) shows stable discharge capacities of ∼0.22–0.25 mAh with Coulombic efficiency approaching ∼100%, confirming good reversibility. However, comparative experiments using Pt‐based counter electrodes, which were predominantly employed throughout this study, produced more uniform MnO_2_ deposition and superior electrochemical performance despite their smaller surface area. These results suggest that counter electrode surface area is not the dominant factor governing deposition quality or electrochemical performance. Instead, the intrinsic electrochemical stability and catalytic activity of Pt provide more favorable deposition conditions. To evaluate the capacity contribution from conductive carbon cloth (CC), control cells were assembled using pristine CC with 2 M ZnSO_4_ and a Zn anode (Figure S19). Galvanostatic charge–discharge measurements indicate negligible capacity contribution from the CC substrate. Overall, these results demonstrate that magnetic‐field‐assisted operation is an effective strategy to enhance electrochemical performance, while also emphasizing the need to carefully balance capacity and long‐term cycling stability in Zn‐MnO_2_ systems.

## 4 Conclusion

This work demonstrates a scalable MED strategy for engineering high‐performance MnO_2_ cathodes for aqueous Zn‐MnO_2_ batteries. The application of an external magnetic field during deposition induces Lorentz‐force‐driven MHD convection, enabling the formation of dense, uniform, and strongly adhered MnO_2_ coatings with enhanced crystallinity and higher mass loading. As a result, MED cathodes deliver superior electrochemical performance, achieving a high initial areal capacity of 0.62 mAh cm^−^^2^, improved capacity retention (73% after 100 cycles), and stable cycling beyond 300 cycles, significantly outperforming ED cathodes. The optimized deposition condition (1.5 V, 20 min MED) provides the best balance between capacity and cycling stability. Furthermore, introducing internal magnetic fields during battery operation reveals a tunable enhancement in electrochemical behavior, with 10 mT identified as the optimal condition for improving Zn^2^^+^ transport and reducing polarization. Importantly, this study establishes that magnetic‐field‐assisted processes not only homogenize electrodeposited material but also enables control over ion transport dynamics, revealing a capacity‐stability trade‐off governed by MHD convection. A comprehensive structural and electrochemical analyses confirm that the enhanced performance is contributed from favorable δ‐phase MnO_2_ through MED and efficient Zn^2^^+^ intercalation, and reduced charge‐transfer resistance derived from internal magnets. Although this study focuses on electrodeposited MnO_2_, the MED concept may be extendable to other cathode systems in which electrodeposition, interfacial ion transport, or concentration polarization limits performance. For example, preformed MnO_2_ cathodes may benefit primarily during battery operation through field modulated electrolyte transport, whereas electrodeposited metal oxides may also benefit during synthesis through MHD controlled nucleation and growth. V‐based cathodes could similarly be influenced if their electrochemical response is limited by Zn^2+^ transport or interfacial polarization. However, the strength of the magnetic field effect will depend on the reaction chemistry, electrolyte composition, electrode geometry, and field orientation; extension to other cathodes requires system specific validation.

## Funding

The authors would also like to acknowledge support from the National Science Foundation under Grant No. 2501275 given to J. G. B.

## Conflicts of Interest

Dr. Jeffrey G. Bell supervised the project. All authors have given approval to the final version of the manuscript.

## Supporting information




**Supporting File 1**: smll74574‐sup‐0001‐SuppMat.docx.

**Supporting File 2**: smll74574‐sup‐0002‐VideoS1.mov.

**Supporting File 3**: smll74574‐sup‐0003‐VideoS2.mov.

## Data Availability

Data will be made available on request.
