# Supplementary material for: Magneto‐Electrodeposition and Field‐Enhanced Ion Transport in Zn‐MnO2 Batteries
Source: Small. 2026 Jul 19;22(50):e74574. doi: 10.1002/smll.74574 (PMC13548947; doi:10.1002/smll.74574)
Supplement: Supplementary file 1 [file SMLL-22-e74574-s002.docx]

**Supporting Information to**

Magneto-Electrodeposition and Field-Enhanced Ion Transport in Zn-MnO_2_ Batteries

Pedaballi Sireesha^1^, William T. McLeod^1^, Kaylie A. McCracken^1^, Jeffrey G. Bell^1,2,*^

^1^ Department of Chemistry, Washington State University, Pullman, Washington, 99164, United States.

^2^ The Gene and Linda Voiland School of Chemical Engineering and Bioengineering, Washington State University, Washington 99164, United States.

* Corresponding Author: jeffrey.g.bell@wsu.edu

**Figure S1.** Overview of the experimental setup for electrodeposition (ED) and magneto-electrodeposition (MED) of MnO₂ cathodes: (a) Schematic of 3D-printed well cell setup components, 3-electrode well-cell configuration used for cathode fabrication, (b) electrodeposition setup, and (c) MED setup with 0.4 T block magnet, both captured during video recording.

**Figure S2***. i-t* curves of electrodeposited and magneto-electrodeposited (MED) MnO_2_ cathodes at varying voltages (1.4-1.7 V) for 10 minutes. These plots are from the average of 3 cells.

**Figure S3**. Evidence of surface SEM images of the pristine carbon cloth (a), MED MnO_2_ (b), and ED MnO_2_ (c) at 1.5 V for 20 minutes.

**Figure S4.** SEM-EDX mapping images of MED MnO₂ fresh cathodes at 1.5 V for 20 minutes.

**Figure S5.** SEM-EDX mapping images of electrodeposited MnO₂ fresh cathodes prepared at different deposition voltages (1.4-1.7 V) for 20 minutes.

**Figure S6.** SEM-EDX mapping images of MED MnO₂ fresh cathodes prepared at different deposition voltages (1.4-1.7 V) for 20 minutes.

**SI Table 1.** Tabulated elemental mapping results of C, O, and Mn wt% for electrodeposited and magneto-electrodeposited cathodes.

**SI Table 2.** Tabulated crystal systems and structural characteristics of MnO₂ polymorphs, with details from the literature for reference.

**Figure S7.** Standard reference patterns for polymorphs of MnO_2_.

**Figure S8**. Crystallographic Models of MnO₂ Polymorphs a) & b) tetragonal) c)cubic, d),e) hexagonal and f) layered hexagonal structures of MnO_2_.

**SI Table 3.** Tabulated EIS fitting results for its corresponding Nyquist plots of ED and MED at different potentials (1.4-1.7 V).

**SI Table 4.** Tabulated EIS fitting results for their corresponding Nyquist plots of 1.5 V for 20 min

electrodeposited and MED cathodes with 1M ZnSO_4_ in Swagelok configuration.

**Figure S9.** Cyclic voltammograms of varying voltage (1.4 -1.7 V) MnO_2_/CC cathodes with (a) and without magnetic fields (b). Experiments were performed in 2 M ZnSO_4_.

**Figure S10.** Effect of magnetic fields on transport kinetics. Panel A) Cyclic voltammograms recorded at different scan rates using carbon cloth and 1 M MnSO_4_-3M H_2_SO_4_ electrolyte a) No magnetic field, and d) Magnetic field applied. Cyclic voltammograms recorded at different scan rates using b) ED and e) MED MnO₂ cathodes in 2 M ZnSO_4_ electrolyte. Panel c) and f) corresponding linear relationship between peak current (i_p_) and the square root of scan rate ($\sqrt{v}$) for Mn^2+^ transport (deposition process) and Zn^2+^ transport (battery operation), respectively.

**Figure S11.** TXRF analysis: Manganese (Mn) content tested for electrodeposited and magneto-electrodeposited MnO₂ cathode samples. These results are from an average of 3 samples.

**Calculation of the g-factor for EPR :**

Equation used to calculate $g$-factor

$$g=\frac{hv}{\beta B}$$

*Where,*

$h=$ Planck constant ($6.626\times{10}^{-34} )$

$v$= microwave frequency (X-band ≈ 9.5 GHz)

$\beta$= Bohr magneton $(9.274\times{10}^{-24})$

$B$= Resonance field (Tesla)

**Figure S12.** Initial cycle galvanic charge-discharge profiles for ED and MED cathodes fabricated at: a) 1.4 V, b) 1.5 V, c) 1.6 V, and d) 1.7 V cycled at a 1.2 C-rate.

**Figure S13.** Galvanostatic charge-discharge cycling performance of ED and MED MnO_2_ cathodes fabricated at different potentials, 1.4 V, 1.6 V, and 1.7 V for 20 min at a 1.2 C-rate. These plots are from the average of 3 cells.

**Figure S14:** Electrochemical performance of MnO₂ cathodes electrodeposited at 1.5 V with and without magnetic field assistance: (a) Cyclic voltammograms at 10 mV/s showing redox behavior, (b) Electrochemical impedance spectra of 1.5 V CC/MnO_2_ cathodes with and without magnetic fields.

**Figure S15.** The coulombic efficiencies of MnO₂ cathodes electrodeposited at 1.5 V with and without magnetic field assistance: (a) ED MnO_2_ cathodes at 1.5 V for different times (2 min to 30 min), (b) MED MnO_2_ cathodes at 1.5 V for different times (2 min to 30 min) at 0.4 mA/cm^2^. These plots are from the average of 3 cells.

**Figure S16.** SEM surface top view and cross-sectional images of Zn anodes recovered after cycling from A) 0 mT and B) 10 mT cells. Inlet provides cross-sectional images of the corresponding anodes.

**Figure S17.** Galvanostatic charge-discharge (GCD) performance of MED MnO₂ cathodes) 1.5 V for 20 min) with different magnetic field strengths, 20 mT and 30mT at 1.2 C. This plot is from an average of 3 cells.

**Figure S18.** Electrochemical evaluation of MED MnO₂ cathodes deposited using a large surface area counter electrode: (a) Chronoamperometric current-time response during magneto-electrodeposition at 1.5 V for 20 min, (b) Self-discharge profile of the resulting Zn-MnO₂ cell, (c) Nyquist plots from electrochemical impedance spectroscopy before and after cycling, and (d) Cycling performance and Coulombic efficiency of the MED MnO₂ cathode. These plots are from the average of 3 cells.

To evaluate the influence of counter electrode surface area on the magneto-electrodeposition (MED) process, MnO₂ cathodes were deposited using the optimized 1.5 V for 20 min protocol with a large surface area carbon cloth counter electrode. The chronoamperometric response shown in Figure.S15(a) exhibits an initially higher current followed by a gradual decay during deposition, which is characteristic of nucleation and growth processes during MnO₂ formation. The slightly elevated current compared to earlier depositions is attributed to the improved charge balance and reduced polarization resulting from the larger counter electrode area, which facilitates more efficient charge compensation and stable ion transport in the electrolyte. The electrochemical behavior of the resulting MED MnO₂ cathode is shown in Figure.S15(b) , where the Zn-MnO₂ cell displays a stable discharge profile with a distinct plateau around ~1.6-1.7 V, indicating reversible Zn²⁺ insertion/extraction within the MnO₂ structure. The Nyquist plots in Figure S15(c) reveal a noticeable reduction in charge transfer resistance after cycling, suggesting improved electrode wetting and activation of electrochemically accessible sites during initial cycles. Furthermore, the cycling performance in Figure D demonstrates stable discharge capacities of ~0.22-0.25 mAh with Coulombic efficiency approaching ~100%, confirming good reversibility and stable electrochemical behavior of the MED MnO₂ cathode fabricated using the large counter electrode configuration. However, comparative experiments using Pt-based counter electrodes ( predominantly used in the entire study), even with significantly smaller surface areas, produced more uniform MnO₂ deposition and superior electrochemical performance relative to the large-area carbon cloth counter electrode. These results indicate that the counter electrode surface area does not play a dominant role in determining the deposition quality or electrochemical performance of MED MnO₂ cathodes, while the intrinsic electrochemical stability and catalytic activity of Pt provide more favorable deposition conditions. Overall, the MED process demonstrates robust and reproducible MnO₂ cathode formation independent of counter electrode surface area, highlighting its reliability for scalable electrode fabrication

**Figure S19.** Galvanostatic charge-discharge cycling performance of pristine carbon cloth (CC) cathode and (a) areal capacity and (b) coulombic efficiency with 2 M ZnSO_4_ and Zn anode at 0.4mA/cm^2^. These plots are from the average of 3 cells.

**Table 5**. Comparative Overview of MnO₂ Synthesis via Various Electrolytes and Methods.

| **Study** | **Electrolyte Composition** | **Deposition Method** | **Magnetic**  **Field** | **MnO₂**  **Phase / Morphology** | **Reference** |  |
| --- | --- | --- | --- | --- | --- | --- |
| Pedaballi et.al | 1M MnSO_4_-3M H₂SO₄ | Magneto-electrodeposition (Potentiostatic) |  | Mixed  (α, β, γ, and δ ) | **This work** |  |
| Mishra et al. | 0.1 M KMnO₄ | Potentiostatic, Potentiodynamic, Galvanostatic |  | ε-MnO₂  porous microstructure | [1] |  |
| Liu et al. | MnSO₄,(NH₄)₂S₂O₈ | Microwave Hydrothermal |  | β-MnO₂ | [2] | |
| Babakhani et al. | 0.01 M MnSO₄  0.01 M Mn(CH₃COO)₂ | Anodic Deposition |  | MnO₂ rods | [3] | |
| Cestaro et al. | 50 mM Mn(CH₃COO)₂  100 mM Na₂SO₄ | Potentiostatic Deposition |  | MnO, Mn₂O₃, Mn₃O₄  (post-calcination) | [4] | |
| Dai et al. | Mn(CH₃COO)₂ | Electrodeposition |  | Nanobelts | [5] | |
| Diantoro et al. | MnSO₄·H₂O with Na₂S₂O₈  AgNO₃, | Hydrothermal |  | MnO₂ nanorods | [6] | |
| [Boytsova](https://link.springer.com/article/10.1134/S0036023615050022#auth-O__V_-Boytsova-Aff1-Aff2) et al. | KMnO₄, NaNO₂,H₂SO₄ | Microwave Hydrothermal |  | Nanocrystalline MnO₂ | [7] | |
| Xiao et al. | MnCl₂,Mn(NO₃)₂, MnSO₄,  Mn(CH₃COO)₂ | Galvanostatic Deposition |  | α-MnO₂ films | [8] | |
| Yi et al. | Mn(CH₃COO)₂ | Anodic Deposition |  | MnO₂ | [9] | |
| Nakayama et al. | 0.01-0.1 M KMnO₄ | Galvanostatic, Pulse, Reverse-Pulse Deposition |  | Nanostructured MnO₂ films | [10] | |
| Tang et al. | (CH_3_COO)_2_Mn·4H_2_O  HNO_3_ | Sol-gel process |  | MnO₂ Nanorods | [11] | |

**Table 6.** Comparative electrochemical performance of MnO₂ based systems and their cyclability.

| **Study** | **Cathode/System** | **Synthesis Strategy** | **Areal Capacity** | **Cycling Stability** | **Magnetic Field?** | **Reference** |
| --- | --- | --- | --- | --- | --- | --- |
| Zhnag et al. | MnO₂ on dual 3D conductive skeletons | Electrodeposition on 3D framework | ~1.3–1.5 mAh cm⁻² | >225 cycles | No | [12] |
| Gao et al. | Polydopamine-coated MnO₂ | Surface coating strategy | ~0.8–1.0 mAh cm⁻² | >500 cycles | No | [13] |
| Campos et al. | Al-doped vacancy-rich MnO₂ | Defect engineering | ~0.9 mAh cm⁻² | >1000 cycles | No | [14] |
| Song et al. | δ-MnO₂ nanosheet cathode | Hydrothermal growth | ~0.6–0.8 mAh cm⁻² | ~300 cycles | No | [15] |
| Baatiyah et al. | Binder-free MnO₂/Carbon Cloth | Electrodeposition | ~0.4–0.7 mAh cm⁻² | 200–400 cycles | No | [16] |
| Kankanallu et al. | Oxygen-deficient MnO₂ arrays | Defect engineering | ~0.8–1.2 mAh cm⁻² | >500 cycles | No | [17] |
| Dai et al. | Hierarchical MnO₂ nanoflakes/CC | Electrodeposition | ~0.5–0.9 mAh cm⁻² | ~200 cycles | No | [18] |
| Zhou et al. | MnO₂ nanowire textile cathode | Hydrothermal + textile support | ~0.7–1.1 mAh cm⁻² | >300 cycles | No | [19] |
| Xue et al. | Zn–MnO₂ under external magnetic field | Commercial MnO₂ cathode | Capacity enhancement reported | Improved cycling | Yes | [20] |
| Pedaballi et al. | MED MnO₂/Carbon Cloth | Magneto-electrodeposition + internal magnets | 0.552 mAh cm⁻² | 315 cycles | Yes | This work |

**References:**

[1]	R.K. Mishra, C.S. Prajapati, R.R. Shahi, A.K. Kushwaha, P.P. Sahay, Influence of electrodeposition modes on the electrochemical performance of MnO2 films prepared using anionic MnO4− (Mn7+) precursor, Ceram. Int. 44 (2018) 5710–5718. https://doi.org/10.1016/j.ceramint.2017.12.224.

[2]	M. Liu, Q. Zhao, H. Liu, J. Yang, X. Chen, L. Yang, Y. Cui, W. Huang, W. Zhao, A. Song, Y. Wang, S. Ding, Y. Song, G. Qian, H. Chen, F. Pan, Tuning phase evolution of β-MnO2 during microwave hydrothermal synthesis for high-performance aqueous Zn ion battery, Nano Energy 64 (2019) 103942. https://doi.org/10.1016/j.nanoen.2019.103942.

[3]	W. Huang, J. Li, Y. Xu, Nucleation/Growth Mechanisms and Morphological Evolution of Porous MnO2 Coating Deposited on Graphite for Supercapacitor, Mater. 10 (2017) 1205. https://doi.org/10.3390/ma10101205.

[4]	R. Cestaro, B. Rheingans, P. Schweizer, A. Müller, C. Vockenhuber, C. Cancellieri, L.P.H. Jeurgens, P. Schmutz, Well‐defined synthesis of crystalline MnO, Mn_2_ O_3_ , and Mn_3_ O_4_ phases by anodic electrodeposition and calcination, J. Am. Ceram. Soc. 107 (2024) 8676–8690. https://doi.org/10.1111/jace.20081.

[5]	X. Dai, M. Zhang, J. Li, D. Yang, Effects of electrodeposition time on a manganese dioxide supercapacitor, RSC Adv. 10 (2020) 15860–15869. https://doi.org/10.1039/D0RA01681K.

[6]	M. Diantoro, I. Istiqomah, Y.A. Fath, N. Nasikhudin, Y. Alias, W. Meevasana, Potential of MnO_2_ ‐based composite and numerous morphological for enhancing supercapacitors performance, Int. J. Appl. Ceram. Technol. 20 (2023) 2077–2098. https://doi.org/10.1111/ijac.14377.

[7]	O.V. Boytsova, T.O. Shekunova, A.E. Baranchikov, Nanocrystalline manganese dioxide synthesis by microwave-hydrothermal treatment, Russ. J. Inorg. Chem. 60 (2015) 546–551. https://doi.org/10.1134/S0036023615050022.

[8]	F. Xiao, Y.L. Xu, Effect of Plating Solutions on Supercapacitor Characteristics of MnO_2_ Films, Adv. Mater. Res. 113–116 (2010) 1810–1813. https://doi.org/10.4028/www.scientific.net/AMR.113-116.1810.

[9]	C.P. Yi, S.R. Majid, The Electrochemical Performance of Deposited Manganese Oxide-Based Film as Electrode Material for Electrochemical Capacitor Application, in: R. Inguanta, C. Sunseri (Eds.), Semicond. - Growth Charact., InTech, 2018. https://doi.org/10.5772/intechopen.71957.

[10]	M. Nakayama, W. Yoshida, Electrodeposited Manganese Dioxides and Their Composites as Electrocatalysts for Energy Conversion Reactions, ChemSusChem 18 (2025) e202401907. https://doi.org/10.1002/cssc.202401907.

[11]	W. Tang, X. Shan, S. Li, H. Liu, X. Wu, Y. Chen, Sol–gel process for the synthesis of ultrafine MnO2 nanowires and nanorods, Mater. Lett. 132 (2014) 317–321. https://doi.org/10.1016/j.matlet.2014.05.211.

[12]	Y. Zhang, S. Luo, G. Yang, L. Yu, S. Ye, C. Jiang, Y. Wu, Electrodeposited ε-MnO2 on carbon fibers as an ideal cathode material for aqueous zinc-ion batteries, Mater. Lett. 361 (2024) 135993. https://doi.org/10.1016/j.matlet.2024.135993.

[13]	Y. Gao, R. Wang, P. Feng, X. Tao, J. Chen, Electrodeposition-engineering MnO2-Mn2O3 dual-phase cathode for binder-free zinc-ion batteries, J. Electroanal. Chem. 1011 (2026) 120084. https://doi.org/10.1016/j.jelechem.2026.120084.

[14]	A.R. Campos, M.N. Vila, M. Haddad, L.M. Housel, E. Takeuchi, A. Marschilok, K. Takeuchi, A.S. Poyraz, Facile Electrodeposition and Aging to Generate 3-Dimensional *α* -MnO_2_ Battery Cathodes, J. Electrochem. Soc. 169 (2022) 100516. https://doi.org/10.1149/1945-7111/ac9648.

[15]	Y. Song, J. Li, R. Qiao, X. Dai, W. Jing, J. Song, Y. Chen, S. Guo, J. Sun, Q. Tan, Y. Liu, Binder-free flexible zinc-ion batteries: one-step potentiostatic electrodeposition strategy derived Ce doped-MnO2 cathode, Chem. Eng. J. 431 (2022) 133387. https://doi.org/10.1016/j.cej.2021.133387.

[16]	B. Baatiyah, Y.S. Wudil, M.A. Gondal, Binder-free electrodeposited Co-MnO2 cathode with enhanced defect chemistry for high-performance aqueous zinc-ion batteries, J. Phys. Chem. Solids 215 (2026) 113727. https://doi.org/10.1016/j.jpcs.2026.113727.

[17]	V.R. Kankanallu, A. Adeniyi, J. Bai, L. Ma, H. Zhong, X. Xiao, Z. Ren, S. Ehrlich, J. Moncada, A. Tayal, E. Stavitski, X. Tong, K.J. Takeuchi, A.C. Marschilok, E.S. Takeuchi, M. Ge, Y.K. Chen-Wiegart, Reaction Mechanism of Electrodeposited ε-MnO_2_ : A Proton-Centered Pathway in Aqueous Zn-Ion Systems, ACS Appl. Mater. Interfaces 18 (2026) 26078–26094. https://doi.org/10.1021/acsami.5c22636.

[18]	X. Dai, M. Zhang, T. Li, X. Cui, Y. Shi, X. Zhu, P. Wangyang, D. Yang, J. Li, Effect of current on electrodeposited MnO2 as supercapacitor and lithium-ion battery electrode, Vacuum 195 (2022) 110692. https://doi.org/10.1016/j.vacuum.2021.110692.

[19]	X. Zhou, S. Chen, Y. Zhang, B. Yu, Y. Chen, Y. Liu, S. Li, L. Liu, H. Jin, J. Deng, Q. Tan, Three‐Dimensional Conductive Interface and Tip Structure of MnO_2_ Electrode Facilitate Superior Zinc Ion Batteries, Small Struct. 5 (2024) 2400057. https://doi.org/10.1002/sstr.202400057.

[20]	X. Xue, S. Chandrasekaran, J.-B. Forien, A. Pinongcos, M. Parra, L. Valdovinos, S. Avalos Franco, B. Dunn, M.A. Worsley, C.A. Orme, Y. Li, Thermodynamic Control of Interface Directs MnO_2_ Nucleation Chemistry for Dense and Conformal Electrodeposition, J. Am. Chem. Soc. 148 (2026) 20830–20840. https://doi.org/10.1021/jacs.6c03948.
